# Identification of *Ligularia* Herbs Using the Complete Chloroplast Genome as a Super-Barcode

**DOI:** 10.3389/fphar.2018.00695

**Published:** 2018-07-03

**Authors:** Xinlian Chen, Jianguo Zhou, Yingxian Cui, Yu Wang, Baozhong Duan, Hui Yao

**Affiliations:** ^1^Key Lab of Chinese Medicine Resources Conservation, State Administration of Traditional Chinese Medicine of the People’s Republic of China, Institute of Medicinal Plant Development, Chinese Academy of Medical Sciences & Peking Union Medical College, Beijing, China; ^2^College of Pharmaceutical Science, Dali University, Dali, China

**Keywords:** *Ligularia* Cass., chloroplast genome, identification, super-barcode, Illumina sequencing

## Abstract

More than 30 *Ligularia* Cass. (Asteraceae) species have long been used in folk medicine in China. Morphological features and common DNA regions are both not ideal to identify *Ligularia* species. As some *Ligularia* species contain pyrrolizidine alkaloids, which are hazardous to human and animal health and are involved in metabolic toxification in the liver, it is important to find a better way to distinguish these species. Here, we report complete chloroplast (CP) genomes of six *Ligularia* species, *L. intermedia*, *L. jaluensis*, *L. mongolica*, *L. hodgsonii*, *L. veitchiana*, and *L. fischeri*, obtained through high-throughput Illumina sequencing technology. These CP genomes showed typical circular tetramerous structure and their sizes range from 151,118 to 151,253 bp. The GC content of each CP genome is 37.5%. Every CP genome contains 134 genes, including 87 protein-coding genes, 37 tRNA genes, eight rRNA genes, and two pseudogenes (*ycf1* and *rps19*). From the mVISTA, there were no potential coding or non-coding regions to distinguish these six *Ligularia* species, but the maximum likelihood tree of the six *Ligularia* species and other related species showed that the whole CP genome can be used as a super-barcode to identify these six *Ligularia* species. This study provides invaluable data for species identification, allowing for future studies on phylogenetic evolution and safe medical applications of *Ligularia*.

## Introduction

*Ligularia* Cass., belonging to the Senecioneae tribe of Asteraceae, comprises about 140 species of perennial herbs. These species are distributed in Asia and Europe, with a total of 123 species distributed in China, 89 of which are endemic (Liu and Illarionova, 1989). In China, *Ligularia* species are mainly distributed in mountainous areas in the southwest (Liu and Illarionova, 1989) and more than 30 *Ligularia* species have long been used in folk medicine (Wang, 2007). The roots, stems, leaves, and flowers of them contain various chemical compounds, such as sesquiterpenes (Wang, 2007; Shimizu et al., 2014; Saito et al., 2017) and alkaloids (Asada et al., 1981; Feng, 2016). They are used as herbal medicines for the treatment of bronchitis, coughing, pulmonary tuberculosis, and hemoptysis. These herbal medicines are usually used as substitutes for Asteris Radix et Rhizoma which originates from *Aster tataricus* L. and is recorded in the Chinese Pharmacopoeia (Lin and Liu, 1989; Chinese Pharmacopoeia Commission, 2015). Approximately, 3% of flowering plants (as many as 6,000 species), including *Ligularia* species (Smith and Culvenor, 1981; Stegelmeier et al., 1999), contain pyrrolizidine alkaloids (PAs). It has been reported that various *Ligularia* species contain PAs, including *L. japonica* (Asada et al., 1981), *L. wilsoniana* (Xiong et al., 2016), *L. duciformis*, *L. intermedia*, *L. hodgsonii*, and *L. veitchiana* (Pu et al., 2004). PAs are phytoalexins that function in plant defense systems against herbivores, insects, and plant pathogens. However, they are harmful to human and animal health (Jank and Rath, 2017; Martinello et al., 2017), as they are involved in metabolic toxification in the liver caused by PA poisoning (Bull et al., 1968; Prakash et al., 1999). The German Federal Department of Health stated that the safe total daily dose of PA is less than 1 μg, and doctors do not allow continuous administration of drugs with PA for more than 6 weeks. In addition, all PA-containing products are banned in Australia (Wiedenfeld and Edgar, 2011).

*Ligularia* has been traditionally classified based on morphological structures, such as the arrangement of inflorescences, leaf shape, leaf veins, and phyllaries (Liu and Illarionova, 1989). Interspecific hybridization of *Ligularia* species is common and their morphological variation is complicated (Hanai et al., 2012; Yu et al., 2014; Saito et al., 2017), making it difficult to correctly identify species. Common DNA barcoding sequences (ITS, *matK*, *psbA-trnH*, and *rbcL*) are also not ideal for identifying *Ligularia* species (He and Pan, 2015). Recently, researchers have screened sequences from the whole chloroplast (CP) genome from numerous plant taxa, such as *Juglans* L. plants and bamboo (Zhang et al., 2011; Hu et al., 2016), or use CP genome as a super-barcode to distinguish species (Xia et al., 2016). The CP genome is highly conserved in plants regardless of the size, structure, or gene content (Tonti-Filippini et al., 2017), and the majority of the retained core genes are involved in the light reactions of photosynthesis or in functions related to transcription or translation (Sato et al., 1999). The CP genome map is a circular DNA molecule that includes a SSC region, a LSC region, and two inverted-repeat (IRa and IRb) regions (Sato et al., 1999). Several CP genomes from Asteraceae have previously been reported, including CP genomes from *Aster* (Choi and Park, 2015), *Ambrosia* (Nagy et al., 2017), *Carthanus* (Lu et al., 2015), and *Taraxacum* (Salih et al., 2017). However, only one CP genome from *Ligularia*, for *L. fischeri*, has previously been published (Lee et al., 2016). In this study, we report the CP genomes of six *Ligularia* species, *L. intermedia*, *L. jaluensis*, *L. mongolica*, *L. hodgsonii*, *L. veitchiana*, and *L. fischeri*, obtained through high-performance Illumina sequencing technology. Our aim is to use the CP genome as a super-barcode for the identification of *Ligularia* species to provide invaluable genetic information for future studies.

## Materials and Methods

### Plant Materials and DNA Extraction

Fresh leaves of *L. intermedia* and *L. fischeri* were collected from Baishan City and Tonghua City, Jilin Province, China, respectively. Fresh leaves of *L. jaluensis* and *L. mongolica* were collected from Yanbian Korean Autonomous Prefecture, Jilin Province. These four species were identified by Prof. Junlin Yu from Tonghua Normal University, Jilin. Fresh leaves of *L. hodgsonii* and *L. veitchiana* were collected from Enshi Tujia and Miao Autonomous Prefecture, Hubei Province, and the Qinling Mountains, Shaanxi Province, respectively. These two samples were identified by Prof. Yulin Lin from the Institute of Medicinal Plant Development (IMPLAD), Chinese Academy of Medical Sciences (CAMS), and Peking Union Medical College (PUMC). The exact GPS coordinates for the collection locations of six *Ligularia* species are listed in Supplementary Table S1. Voucher specimens were deposited in the herbarium at IMPLAD. Collected fresh leaves were stored in a -80°C freezer until further use. DNA extraction was performed using a DNeasy Plant Mini Kit (Qiagen Co., Germany) following the manufacturer’s protocol.

### Illumina Sequencing and Genome Assembly

Approximately 5–10 μg of high-quality DNA were used to build shotgun libraries with insert sizes of 500 bp and were sequenced in accordance with the protocol for Illumina Hiseq X technology. The total raw data of the six species were produced with 150 bp paired-end read lengths. The software Trimmomatic (Bolger et al., 2014) was employed to filter low-quality reads from the raw data. After filtering for quality sequences, the remaining clean reads were used to assemble the CP genome sequences. The CP sequences of all plants downloaded from the National Center for Biotechnology Information (NCBI) were used to create a reference database. Then, the clean sequences were mapped to the database and the mapped reads were extracted on the basis of coverage and similarity. The extracted reads were assembled into contigs using SOAPdenovo2 (Luo et al., 2012). The scaffold of the CP genome was constructed using SSPACE (Boetzer et al., 2011), and the gaps were filled using GapFiller (Boetzer and Pirovano, 2012).

### Validation, Annotation, and Sequence Submission

The accuracy of the assembly of the four boundaries (SSC, LSC, IRa, and IRb regions) of the CP sequences was confirmed through PCR and Sanger sequencing using validated primers (Supplementary Table S2). The thermocycler conditions for the PCR were as follows: 94°C for 5 min; 94°C for 30 s, 56°C for 30 s, 72°C for 1.5 min, and 32 cycles; 72°C for 10 min. The online programs Dual Organellar GenoMe Annotator (DOGMA) (Wyman et al., 2004) and CPGAVAS (Liu et al., 2012) were used for the initial annotation of the CP genomes of the six species, followed by manual correction. The complete data from the study were submitted to NCBI under the BioProject ID PRJNA400300 and BioSample ID SAMN07562669. The assembled complete CP genome sequences of the six *Ligularia* species were submitted to NCBI GenBank with the accession numbers MF539929-MF539933, and MG729822.

### Genome Structure Analysis

The software tRNAscan-SE (Schattner et al., 2005) and DOGMA (Wyman et al., 2004) were used to identify tRNA genes. Gene maps were generated using Organellar Genome DRAW v1.2 (Lohse et al., 2007) with default settings and then the gene maps were checked manually. MEGA 6.0 was used to calculate the GC content (Tamura et al., 2013). REPuter (University of Bielefeld, Bielefeld, Germany) (Kurtz et al., 2001) was used to identify the size and location of repeat sequences in the CP genomes of the six *Ligularia* species. We used the MISA software Misa-Microsatellite Identification Tool, 2017^1^ to detect SSRs with the parameter settings the same as those described in Li et al. (2013). All the repeated sequences were manual verified and excess data were removed. The distribution of codon usage was studied using CodonW with the relative synonymous codon usage (RSCU) ratio (Sharp and Li, 1987). The online program Predictive RNA Editor for Plants suite (Mower, 2009) with a cutoff value of 0.8 were used to predict RNA editing sites in the six CP genomes of *Ligularia* species.

### Phylogenetic Analysis

For identification purposes and to further phylogenetic research on this genus, we used mVISTA (Thompson et al., 1994) to compare six *Ligularia* species with *L. hodgsonii* as the reference genome. MEGA 6.0 was used to construct the phylogenetic tree with *Platycodon grandiflorus* and *Adenophora remotiflora* included as outgroups based on ML analysis. The details of the selected species (excluding the six *Ligularia* species) are presented in Supplementary Table S3.

## Results and Discussion

### CP Genome Structure of Six *Ligularia* Species

**FIGURE 1:**
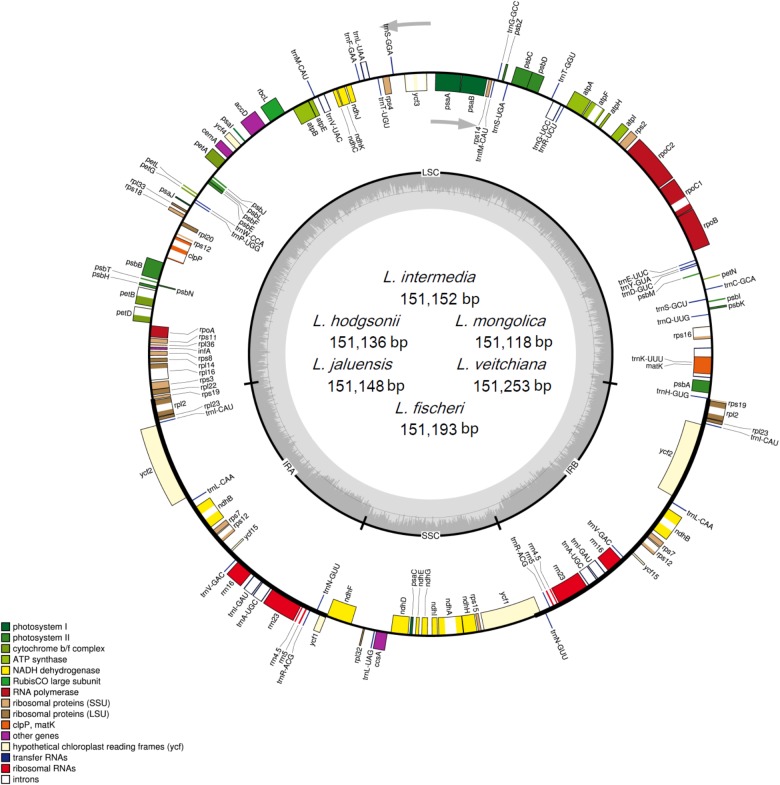
Gene map of the complete CP genomes of the six *Ligularia* species. Genes on the inside of the circle are transcribed clockwise, while those outside are transcribed counter clockwise. The darker gray in the inner circle corresponds to GC content, whereas the lighter gray corresponds to AT content.

The raw data from the six *Ligularia* species is 9.1 Gb for *L. intermedia*, 7.2 Gb for *L. hodgsonii*, 7.4 Gb for *L. jaluensis*, 6.4 Gb for *L. mongolica*, 6.3 Gb for *L. veitchiana*, and 6.2 Gb for *L. fischeri*. The sizes of the six CP genomes range from 151,118 bp for *L. mongolica* to 151,253 bp for *L. veitchiana*, which are similar to other Asteraceae CP genomes (Liu et al., 2013; Salih et al., 2017; Wang et al., 2017; Zhang et al., 2017). The investigated genomes showed typical circular tetramerous structure, including an SSC region and an LSC region, separated by two IR regions (**Figure 1**). The corresponding lengths of the four regions from the six species are similar: the SSC lengths range from 18,214 to 18,247 bp, the LSC lengths range from 83,244 to 83,330 bp, and the IR lengths range from 24,830 to 24,838 bp (**Table 1**). The size of the previously published *L. fischeri* CP genome is 151,133 bp, and included an SSC region (18,233 bp), an LSC region (83,238 bp), and two IR regions (24,831 bp apart) (Lee et al., 2016). Our results showed that all six of the newly sequenced CP genomes have a GC content of 37.5%, which is lower than some Asteraceae species (Liu et al., 2013; Salih et al., 2017; Wang et al., 2017; Zhang et al., 2017). The GC content of four homologous regions of the six CP genomes is the same. However, the distribution of the GC content in each region is uneven. The GC content in the IR region is the largest (43.0%), followed by the LSC region (35.6%), and the region with the lowest GC content is the SSC region (30.7%). Our analysis showed that the high GC content in the IR region is attributed to four rRNA genes (*rrn16*, *rrn23*, *rrn4.5*, and *rrn5*). The AT content of the first, second, and third position of protein-coding genes in the six CP genomes are 54.5–54.6%, 61.9–62.0%, and 70.1%, respectively. The higher AT content in the third site has also been observed in other plants (Yi and Kim, 2012; He et al., 2017; Zhou et al., 2017) and is usually used to distinguish DNA of CP, nucleus, and mitochondria origin (Clegg et al., 1994).

**Table 1:** Summary statistics for assembly of the six CP genomes of *Ligularia* species.

Species names	*L. intermedia*	*L. hodgsonii*	*L. jaluensis*	*L. mongolica*	*L. veitchiana*	*L. fischeri*
Raw reads	53,871,796	42,199,336	43,781,502	37,478,124	36,990,248	36,684,984
Clean reads	52,907,406	41,086,606	42,940,766	36,283,722	35,813,466	35,451,926
Mapped CP reads	434,480	623,388	516,254	378,838	278,424	361,650
Size (bp)	151,152	151,136	151,148	151,118	151,253	151,193
LSC length (bp)	83,258	83,253	83,263	83,244	83,330	83,301
SSC length (bp)	18,232	18,217	18,225	18,214	18,247	18,232
IR length (bp)	24,831	24,833	24,830	24,830	24,838	24,830
Coding (bp)	90,247	90,253	90,247	90,250	90,253	90,247
Non-coding (bp)	60,905	60,883	60,901	60,868	60,000	60,946

Each of the six CP genomes contains 134 genes, including 87 protein-coding genes, 37 tRNA genes, eight rRNA genes, and two pseudogenes (*ycf1* and *rps19*; **Table 2**). Seven protein-coding genes (*ndhB*, *rp12*, *rp123*, *rps12*, *rps7*, *ycf15*, and *ycf2*), seven tRNAs (*trnA-UGC*, *trnI-CAU*, *trnI-GAU*, *trnL-CAA*, *trnN-GUU*, *trnR-ACG*, and *trnV-GAC*), and all of the rRNAs (*rrn16*, *rrn23*, *rrn4.5*, and *rrn5*) are duplicated in the IR regions, which is similar to *Artemisia annua* (Shen et al., 2017) and *Artemisia frigida* (Liu et al., 2013). The CP genomes of the six *Ligularia* species contain a small 3.4 kb inversion within a large 23 kb inversion in the LSC region, which is a unique feature in Asteraceae (Kim et al., 2005; Liu et al., 2013). The LSC region included 62 protein-coding genes and 22 tRNA genes. The SSC region included 11 protein-coding genes and one tRNA gene (*trnL-UAG*). The CP genomes of each of these six *Ligularia* species did not have an inverted SSC region, which has also been found in the CP genomes of *A. frigida* (Liu et al., 2013), *Scutellaria baicalensis* (Jiang et al., 2017), *Carthamus tinctorius* (Lu et al., 2015), and *Juglans* L. (Hu et al., 2016). In contrast, the SSC regions of *Helianthus annuus*, *Lactuca sativa* (Timme et al., 2007), and *Aster spathulifolius* (Choi and Park, 2015) are inverted. The functional *ycf1* copy is located in the IRb-SSC boundary and the pseudogene *ycf1* copy is located in the IRa region. The functional *rps19* copy is on the boundary of LSC and IRa and the pseudogene *rps19* copy is on the IRb region. The coding region occupied 59.67–59.72% of the CP genomes of six *Ligularia* species, including protein-coding genes, tRNA genes, and rRNA genes. Meanwhile, non-coding regions, including introns, pseudogenes, and intergenic spacers occupied 40.28–40.33% of the CP genomes of the six *Ligularia* species.

**Table 2:** List of genes found in the six CP genomes of *Ligularia* species.

No.	Group of genes	Gene names	Amount
1	Photosystem I	*psaA*, *psaB*, *psaC*, *psaI*, *psaJ*	5
2	Photosystem II	*psbA*, *psbB*, *psbC*, *psbD*, *psbE*, *psbF*, *psbH*, *psbI*, *psbJ*, *psbK*, *psbL*, *psbM*, *psbN*, *psbT*, *psbZ*	15
3	Cytochrome b/f complex	*petA*, *petB^∗^*, *petD^∗^*, *petG*, *petL*, *petN*	6
4	ATP synthase	*atpA*, *atpB*, *atpE*, *atpF^∗^*, *atpH*, *atpI*	6
5	NADH dehydrogenase	*ndhA^∗^*, *ndhB^∗^* (×2), *ndhC*, *ndhD*, *ndhE*, *ndhF*, *ndhG*, *ndhH*, *ndhI*, *ndhJ*, *ndhK*	11
6	RubisCO large subunit	*rbcL*	1
7	RNA polymerase	*rpoA*, *rpoB*, *rpoC1^∗^*, *rpoC2*	4
8	Ribosomal proteins (SSU)	*rps2*, *rps3*, *rps4*, *rps7* (×2), *rps8*, *rps11*, *rps12^∗∗^* (×2), *rps14*, *rps15*, *rps16^∗^*, *rps18*, *rps19*	12
9	Ribosomal proteins (LSU)	*rpl2^∗^* (×2), *rpl14*, *rpl16^∗^*, *rpl20*, *rpl22*, *rpl23* (×2), *rpl32*, *rpl33*, *rpl36*	9
10	Other genes	*accD*, *clpP^∗∗^*, *matK*, *ccsA*, *cemA*, *infA*	6
11	Proteins of unknown function	*ycf1*, *ycf2* (×2), *ycf3^∗∗^*, *ycf4*, *ycf15*	5
12	Transfer RNAs	*37 tRNAs* (6 contain an intron, 7 in the IRs)	
13	Ribosomal RNAs	*rrn4.5* (×2), *rrn5* (×2), *rrn16* (×2), *rrn23* (×2)	

*One or two asterisks after genes indicate that gene contains one or two introns, respectively.*

### Codon Usage and RNA Editing Sites

**FIGURE 2:**
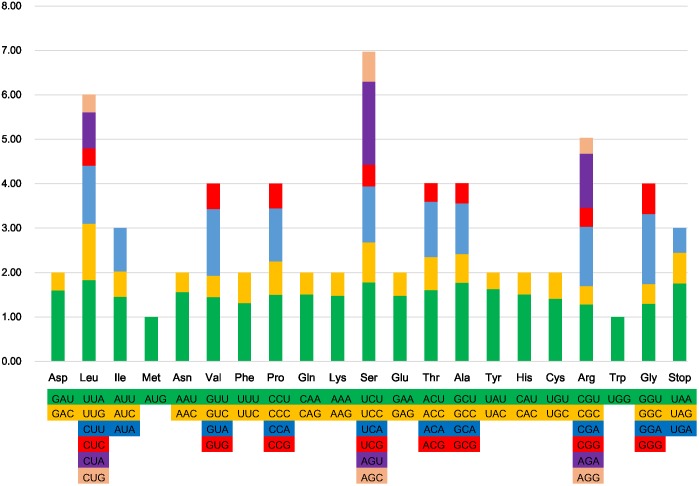
Codon content of 20 amino acid and stop codons in all protein-coding genes of the CP genome of *L. hodgsonii*.

All protein-coding genes in the six *Ligularia* CP genomes are composed of 26,136–26,138 codons. The most and least universal amino acids of the CP genomes of the six *Ligularia* species are leucine (10.8%) and cysteine (1.1%), respectively (**Figure 2**). This is also similar to the CP genome from artichoke (Curci et al., 2015). However, the most universal amino acid from *A. frigida* is isoleucine (Liu et al., 2013). The most and the least abundant amino acids in the *Taraxacum obtusifrons* and *Taraxacum amplum* CP genomes are serine and methionine (Salih et al., 2017), respectively. **Figure 2** shows that with the increase of specific amino acid codes the RSCU increases accordingly. Most of the amino acid codons have preferences, except for methionine and tryptophan. Potential RNA editing sites were predicted for 35 genes from the CP genomes of the six *Ligularia* species. Forty-eight RNA editing sites were identified. S to L of amino acid change appeared most frequently, while R to W and T to I occurred least. Each corresponding gene from the RNA editing sites of the six *Ligularia* species is at the same nucleotide position (Supplementary Table S4).

A total of 18 genes containing introns, including 12 protein-coding genes (*atpF*, *clpP*, *ndhA*, *ndhB*, *petB*, *petD*, *rpl16*, *rpl2*, *rpoC1*, *rps12*, *rps16*, and *ycf3*), and six tRNA genes (*trnA-UGC*, *trnG-UCC*, *trnI-GAU*, *trnK-UUU*, *trnL-UAA*, and *trnV-UAC*; Supplementary Table S5), were identified in this study. Nine protein-coding genes contain only one intron and three protein-coding genes (*clpP*, *rps12*, and *ycf3*) contain two introns. All six tRNAs contain only one intron. *TrnK-UUU* has the longest intron (2,556 bp), which contains *matK*. The *clpP* gene and *ycf3* gene are both located in the LSC region. The *rps12* gene is a special trans-splicing gene with the 5′ exon located in the LSC region, but the 3′ exon located in the IR region. This condition exists in many species, such as *A. frigida* (Liu et al., 2013), artichoke (Curci et al., 2015), and *Aster spathulifolius* (Choi and Park, 2015).

### Long Repeats and SSRs in the CP Genomes From the Six *Ligularia* Species

**FIGURE 3:**
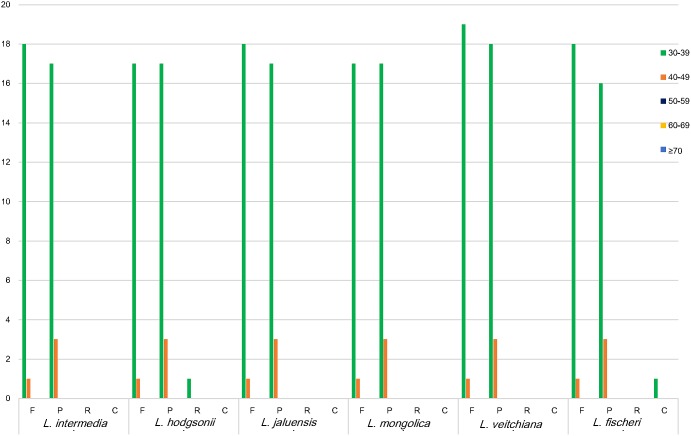
Repeat sequences in six CP genomes. REPuter was used to identify repeat sequences with length ≥30 bp and sequences identified ≥90% in the CP genomes. F, P, R, and C indicate the repeat types F (forward), P (palindrome), R (reverse), and C (complement). Repeats with different lengths are indicated in different colors.

Repeat sequences, which are related to plastome organization (Salih et al., 2017), are mostly distributed in intergenic regions and intron regions, and only a small fraction is present in the genetic region. Four types of long repeats were observed in the CP genomes of the six *Ligularia* species, including forward, palindromic, reverse, and complement repeats (**Figure 3**). The length of the repeat unit ranged from 30 to 48 bp. *Ligularia intermedia* and *L. jaluensis* both had 19 forward and 20 palindromic repeats. *Ligularia hodgsonii* had the following repeats: 18 forward, 20 palindromic, and one reverse. *Ligularia mongolica* had 18 forward and 20 palindromic repeats. *Ligularia veitchiana* had 20 forward and 21 palindromic repeats. *Ligularia fischeri* had the following repeats: 19 forward, 19 palindromic, and one complement. The long repeat sequences observed in the CP genomes of the six *Ligularia* species, with *L. hodgsonii* as the reference, are presented in Supplementary Table S6.

Simple sequence repeats, also called microsatellites, exist widely in the genome, and the sequences consist of one to six nucleotide repeat units (Powell et al., 1995). SSRs are widely used in studies on species identification, population genetics, and phylogenetic studies based on polymorphisms (Yang et al., 2011; Jiao et al., 2012; Xue et al., 2012). Four types of SSRs were found in the CP genomes from the six *Ligularia* species: mononucleotide (56.6–60.7%), dinucleotide (11.5–13.2%), trinucleotide (9.3–9.8%), and tetranucleotide (18.0–21.6%); the SSRs were mainly distributed in the non-coding region of the LSC and SSC. Of all these SSRs, the number of mononucleotide SSRs (A/T) is the largest, ranging from 29 in *L. hodgsonii* to 37 in *L. veitchiana*, enriching A and T in the CP genomes. The next most common SSR is dinucleotide (AT/AT), six dinucleotide SSRs in CP genomes of *L. hodgsonii* and *L. mongolica* and seven dinucleotide SSRs in other four CP genomes. All of the CP genomes from the six species have two trinucleotide AAG/CTT SSRs, one ATC/ATG trinucleotide SSR, and 11 tetranucleotide SSRs (**Table 3**). The CP genome of *L. veitchiana* has three AAT/ATT trinucleotide SSRs, while the other five species only have two trinucleotide SSRs.

**Table 3:** The SSR types of the six CP genomes of *Ligularia* species.

SSR type	Repeat unit	Amount					
		*L. intermedia*	*L. hodgsonii*	*L. jaluensis*	*L. mongolica*	*L. veitchiana*	*L. fischeri*
Mono	A/T	30	29	30	30	37	31
Di	AT/AT	7	6	7	6	7	7
Tri	AAG/CTT	2	2	2	2	2	2
Tri	AAT/ATT	2	2	2	2	3	2
Tri	ATC/ATG	1	1	1	1	1	1
Tetra	AAAG/CTTT	1	1	1	1	1	1
Tetra	AAAT/ATTT	6	6	6	6	6	6
Tetra	AACT/AGTT	1	1	1	1	1	1
Tetra	AATC/ATTG	1	1	1	1	1	1
Tetra	AATT/AATT	1	1	1	1	1	1
Tetra	AGAT/ATCT	1	1	1	1	1	1

### Identification and Phylogenetic Analysis of *Ligularia* Species

The CP genomes from the six *Ligularia* species are highly similar. Among the few variations, non-coding regions exhibited higher levels of variability than the coding regions. The largest change in gene length occurred in pseudogene *ycf1*, with 5,097 bp in *L. mongolica*, 5,100 bp in *L. hodgsonii* and *L. veitchiana*, and 5,094 bp in the other three species. This difference led to a divergence in the length of the coding regions of the six species. The IR regions of the six CP genomes are conservative regardless of the number and order of the genes. Previous research screened highly variable region from CP genomes as the potential DNA barcodes for authenticating species, such as *Dioscorea* (Ma et al., 2018) and *Fritillaria* species (Li et al., 2016).

**FIGURE 4:**
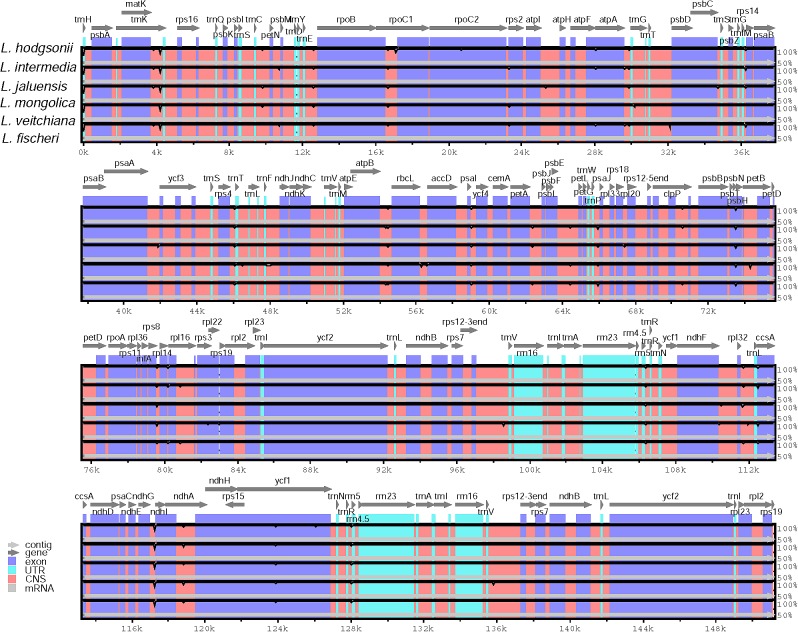
Sequence identity plot comparing six CP genomes with *L. hodgsonii* as a reference using mVISTA. Gray arrows and thick black lines above the alignment indicate genes with their orientation and the position of the IRs, respectively. A cutoff of 70% identity was used for the plots, and the *Y*-scale represents the percent identity ranging from 50 to 100%.

Sequence homology was investigated compared with the reference CP genome from *L. hodgsonii* using the mVISTA software (**Figure 4**). Our results showed high similarity among all sequences. Differences were observed in the intergenic regions of *matK-trnK* and *ndhG-ndhI* (**Figure 4**). There was only one variable site in the *matK-trnK* region and five variable sites in *ndhG-ndhI* region, but this is not enough to distinguish among the six *Ligularia* species. Because of the highly conservative sequences, the structure, and size of the CP genomes of *Ligularia* species, no obvious hypervariable region was screened. Thus, the complete CP genomes were considered to distinguish *Ligularia* species.

**FIGURE 5:**
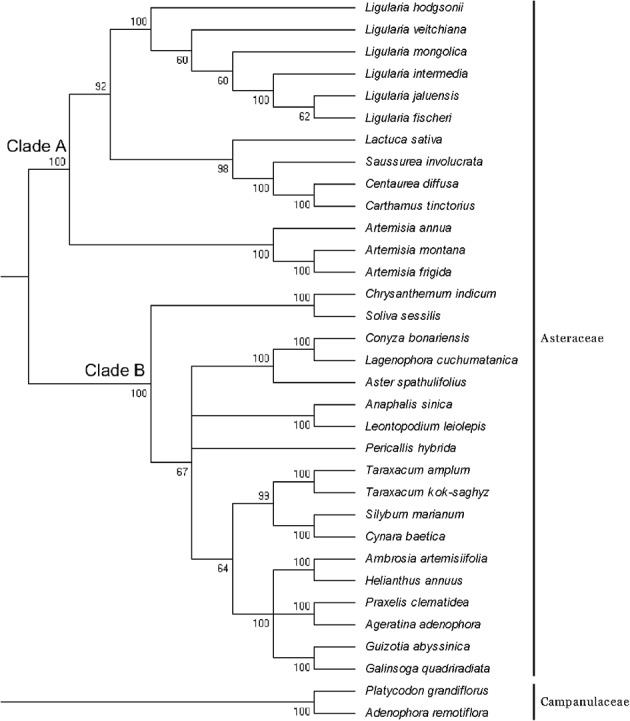
Phylogenetic tree constructed using ML based on complete CP genomes of six *Ligularia* and other 25 species. Numbers above the branches are bootstrap support values.

In addition to the six CP genomes sequenced in this study, 25 other CP genomes from Asteraceae were chosen to construct the phylogenetic tree, and *P. grandiflorus* and *A. remotiflora* (Campanulaceae) were included as outgroups (**Figure 5**). In the ML tree, we identified two main clades (clade A and B) excluding outgroup species. Six species of *Ligularia* were a monophyly with well-supported (100%). The support values in clade A were not less than 60%, and *L. fischeri* and *L. jaluensis* have a close relationship. *Ligularia* is most closely related to *L. sativa*, *Saussurea involucrata*, *Centaurea diffusa*, and *Carthamus tinctorius*. The results showed that the CP genomes can be used to identify the six *Ligularia* species.

## Conclusion

This study reported the CP genomes from six *Ligularia* species, and the structure and composition of the CP genomes are highly similar. Like most Asteraceae species, the CP genomes of the six *Ligularia* species had a small 3.4 kb inversion within a large 23 kb inversion in the LSC region. The ML tree showed that the CP genome can be used to identify the six *Ligularia* species and is expected to become a super-barcode for the identification of *Ligularia* species.

## Author Contributions

HY conceived the study and acquired the funding. XC, YW, and BD collected samples and conducted the experiment. JZ and YC performed the genome assembly and analysis on the data. XC and JZ wrote the manuscript. All authors have read and approved the final manuscript.

## Conflict of Interest Statement

The authors declare that the research was conducted in the absence of any commercial or financial relationships that could be construed as a potential conflict of interest.

## Footnotes

1 http://pgrc.ipk-gatersleben.de/misa/

## Abbreviations

IR: inverted repeat
LSC: large single-copy
ML: maximum likelihood
SSC: small single-copy
SSRs: simple sequence repeats
