# Supplementary material for: Identification of Ligularia Herbs Using the Complete Chloroplast Genome as a Super-Barcode
Source: Front Pharmacol. 2018 Jul 3;9:695. doi: 10.3389/fphar.2018.00695 (PMC6043804; doi:10.3389/fphar.2018.00695)
Supplement: Supplementary file 1 [file Table_1.docx]

Supplementary Material

# TABLE S1 | The exact GPS coordinates for the collection locations of six *Ligularia* species.

| Species names | Collection location | GPS coordinates |
| --- | --- | --- |
| L. intermedia | Baishan City, Jilin Province, China | N 41°25′26″, E 128°12′3″ |
| *L. hodgsonii* | Enshi Tujia and Miao Autonomous Prefecture, Hubei Province, China | N 30°17′42″, E 108°56′10″ |
| *L. jaluensis* | Yanbian Korean Autonomous Prefecture, Jilin Province, China | N 43°06′59″, E 128°53′52″ |
| *L. mongolica* | Yanbian Korean Autonomous Prefecture, Jilin Province, China | N 43°06′59″, E 128°53′52″ |
| *L. veitchiana* | Qinling Mountains, Shaanxi Province, China | N 35°23′52″, E 109°11′16″ |
| *L. fischeri* | Tonghua City, Jilin Province, China | N 41°43′52″, E 125°56′22″ |
