# Supplementary material for: Identification of Ligularia Herbs Using the Complete Chloroplast Genome as a Super-Barcode
Source: Front Pharmacol. 2018 Jul 3;9:695. doi: 10.3389/fphar.2018.00695 (PMC6043804; doi:10.3389/fphar.2018.00695)
Supplement: Supplementary file 2 [file Table_2.docx]

Supplementary Material

# TABLE S2 | Validated primers for confirming the four boundaries of the CP genomes from six *Ligularia* species.

| Regions | Forward/Reverse | Base composition of primers (5'→3') |
| --- | --- | --- |
| LSC-IRb | 25IRbF | TTCTTCGTAGGAACGCCCAC |
|  | 25IRbR | GGGTGCTGTAGCGAAACTGA |
| IRb-SSC | 25SF | CTCTTCCTTCTCCGAGCTCAC |
|  | 25SR | TTTCAGTTAGTATAGCTTCTTTCGG |
|  | 01SF | TCTCCGAGCTCACGTTATGG |
|  | 01SR | TTCAGTTAGTATAGCTTCTTTCGG |
| SSC-IRa | 25IRaF | CAAAAGTTGACAAACTGGGAGGA |
|  | 25IRaR | CCGAGCTCACGTTATGGAAGA |
|  | 01IRaF | GTTGACAAACTGGGAGGATAGGT |
|  | 01IRaR | TCTCCGAGCTCACGTTATGG |
| IRa-LSC | 25LF | CCGATATGCCCTTAGGCACG |
|  | 25LR | GGTTCGATTCGCGTCTTTTCT |
|  | 01LF | CAATTAGCTAGAGCAGCGGG |
|  | 01LR | ATTTGGTTCGATTCGCGTCTT |
