# Supplementary material for: Identification of Ligularia Herbs Using the Complete Chloroplast Genome as a Super-Barcode
Source: Front Pharmacol. 2018 Jul 3;9:695. doi: 10.3389/fphar.2018.00695 (PMC6043804; doi:10.3389/fphar.2018.00695)
Supplement: Supplementary file 3 [file Table_3.docx]

Supplementary Material

# TABLE S3 | Details of the species selected for the ML tree (excluding six *Ligularia* species).

| **Latin name** | **Family** | **GenBank accession number** |
| --- | --- | --- |
| *Ageratina adenophora* | Asteraceae | NC_015621 |
| *Ambrosia artemisiifolia* | Asteraceae | NC_035875 |
| *Anaphalis sinica* | Asteraceae | NC_034648 |
| *Artemisia frigida* | Asteraceae | NC_020607 |
| *Artemisia montana* | Asteraceae | NC_025910 |
| *Artemisia annua* | Asteraceae | NC_034683 |
| *Aster spathulifolius* | Asteraceae | NC_027434 |
| *Carthamus tinctorius* | Asteraceae | KX822074 |
| *Centaurea diffusa* | Asteraceae | NC_024286 |
| *Chrysanthemum indicum* | Asteraceae | NC_020320 |
| *Conyza bonariensis* | Asteraceae | NC_035884 |
| *Cynara baetica* | Asteraceae | NC_028005 |
| *Galinsoga quadriradiata* | Asteraceae | NC_031853 |
| *Guizotia abyssinica* | Asteraceae | EU549769 |
| *Helianthus annuus* | Asteraceae | DQ383815 |
| *Lactuca sativa* | Asteraceae | AP007232 |
| *Lagenophora cuchumatanica* | Asteraceae | KX063879 |
| *Leontopodium leiolepis* | Asteraceae | NC_027835 |
| *Pericallis hybrid* | Asteraceae | NC_031898 |
| *Praxelis clematidea* | Asteraceae | NC_023833 |
| *Saussurea involucrata* | Asteraceae | NC_029465 |
| *Silybum marianum* | Asteraceae | NC_028027 |
| *Soliva sessilis* | Asteraceae | NC_034851 |
| *Taraxacum amplum* | Asteraceae | NC_031816 |
| *Taraxacum kok-saghyz* | Asteraceae | NC_032057 |
| *Adenophora remotiflora* | Campanulaceae | NC_026999 |
| *Platycodon grandiflorus* | Campanulaceae | KX352464 |
