# Supplementary material for: Identification of Ligularia Herbs Using the Complete Chloroplast Genome as a Super-Barcode
Source: Front Pharmacol. 2018 Jul 3;9:695. doi: 10.3389/fphar.2018.00695 (PMC6043804; doi:10.3389/fphar.2018.00695)
Supplement: Supplementary file 4 [file Table_4.docx]

Supplementary Material

# TABLE S4 | RNA editing predicted in the CP genomes from the six *Ligularia* species using the PREP program.

| Gene | Nucleotide Position of six *Ligularia* species | Codon change | Amino acid change | Score |
| --- | --- | --- | --- | --- |
| *acc*D | 451 | CAC-TAC | H-Y | 1 |
|  | 824 | TCG-TTG | S-L | 0.8 |
|  | 1225 | CCA-TCA | P-S | 1 |
|  | 1433 | CCT-CTT | P-L | 1 |
| *atp*A | 773 | TCA-TTA | S-L | 1 |
|  | 791 | CCC-CTC | P-L | 1 |
| *atp*B | - | - | - | - |
| *atp*F | - | - | - | - |
| *atp*I | 629 | TCA-TTA | S-L | 1 |
| *ccs*A | 110 | CCA-CTA | P-L | 0.86 |
|  | 370 | CCC-TCC | P-S | 0.86 |
| *clp*P | - | - | - | - |
| *mat*K | 284 | TCT-TTT | S-F | 0.86 |
|  | 637 | CAT-TAT | H-Y | 1 |
|  | 1240 | CAT-TAT | H-Y | 1 |
| *ndh*A | 566 | TCA-TTA | S-L | 1 |
|  | 1073 | TCC-TTC | S-F | 1 |
| *ndh*B | 149 | TCA-TTA | S-L | 1 |
|  | 467 | CCA-CTA | P-L | 1 |
|  | 586 | CAT-TAT | H-Y | 1 |
|  | 611 | TCA-TTA | S-L | 0.8 |
|  | 737 | CCA-CTA | P-L | 1 |
|  | 746 | TCT-TTT | S-F | 1 |
|  | 830 | TCA-TTA | S-L | 1 |
|  | 836 | TCA-TTA | S-L | 1 |
|  | 1481 | CCA-CTA | P-L | 1 |
| *ndh*D | 2 | ACG-ATG | T-M | 1 |
|  | 383 | TCA-TTA | S-L | 1 |
|  | 599 | TCA-TTA | S-L | 1 |
|  | 878 | TCA-TTA | S-L | 1 |
|  | 887 | CCC-CTC | P-L | 1 |
|  | 1310 | TCA-TTA | S-L | 0.8 |
| *ndh*F | 290 | TCA-TTA | S-L | 1 |
|  | 1340 | TCT-TTT | S-F | 1 |
| *ndh*G | 166 | CAT-TAT | H-Y | 0.8 |
|  | 314 | ACA-ATA | T-I | 0.8 |
| *pet*B | 418 | CGG-TGG | R-W | 1 |
|  | 611 | CCA-CTA | P-L | 1 |
| *pet*D | - | - | - | - |
| *pet*G | - | - | - | - |
| *pet*L | - | - | - | - |
| *psa*B | - | - | - | - |
| *psa*I | - | - | - | - |
| *psb*B | - | - | - | - |
| *psb*E | - | - | - | - |
| *psb*F | 77 | TCT-TTT | S-F | 1 |
| *psb*L | 2 | ACG-ATG | T-M | 1 |
| *rpl*20 | 308 | TCA-TTA | S-L | 0.86 |
| *rpl*23 | - | - | - | - |
| *rpl*2 | - | - | - | - |
| *rpo*A | 824 | TCA-TTA | S-L | 1 |
| *rpo*B | 983 | GCG-GTG | A-V | 1 |
| *rpo*C1 | 511 | CCC-TCC | P-S | 1 |
|  | 1592 | GCA-GTA | A-V | 0.86 |
|  | 2045 | CCC-CTC | P-L | 1 |
| *rpo*C2 | 2701 | CAT-TAT | H-Y | 1 |
|  | 3701 | TCG-TTG | S-L | 0.86 |
| *rps*14 | 80 | TCA-TTA | S-L | 1 |
|  | 149 | CCA-CTA | P-L | 1 |
| *rps*16 | - | - | - | - |
| *rps*2 | 248 | TCA-TTA | S-L | 1 |
| *rps*8 | - | - | - | - |
| *ycf*3 | - | - | - | - |
