# Supplementary material for: Identification of Ligularia Herbs Using the Complete Chloroplast Genome as a Super-Barcode
Source: Front Pharmacol. 2018 Jul 3;9:695. doi: 10.3389/fphar.2018.00695 (PMC6043804; doi:10.3389/fphar.2018.00695)
Supplement: Supplementary file 5 [file Table_5.docx]

Supplementary Material

# TABLE S5 | The length and location of the introns in the CP genomes of the six *Ligularia* species.

| *L. intermedia* | | | | | | |
| --- | --- | --- | --- | --- | --- | --- |
| Gene | Location | Exon I (bp) | Intron I (bp) | Exon II (bp) | Intron II (bp) | Exon III (bp) |
| *atpF* | LSC | 145 | 708 | 410 |  |  |
| *clpP* | LSC | 71 | 817 | 291 | 621 | 229 |
| *ndhA* | SSC | 553 | 1063 | 539 |  |  |
| *ndhB* | IR | 777 | 671 | 756 |  |  |
| *petB* | LSC | 6 | 762 | 642 |  |  |
| *petD* | LSC | 9 | 718 | 474 |  |  |
| *rpl16* | LSC | 9 | 1035 | 399 |  |  |
| *rpl2* | IR | 393 | 667 | 435 |  |  |
| *rpoC1* | LSC | 432 | 721 | 1641 |  |  |
| *rps12* | LSC | 114 | - | 232 | 530 | 26 |
| *rps16* | LSC | 41 | 841 | 214 |  |  |
| *trnA-UGC* | IR | 38 | 821 | 35 |  |  |
| *trnG-UCC* | LSC | 23 | 727 | 47 |  |  |
| *trnI-GAU* | IR | 42 | 772 | 35 |  |  |
| *trnK-UUU* | LSC | 37 | 2556 | 35 |  |  |
| *trnL-UAA* | LSC | 37 | 440 | 50 |  |  |
| *trnV-UAC* | LSC | 38 | 573 | 37 |  |  |
| *ycf3* | LSC | 125 | 697 | 229 | 740 | 153 |
| *L. hodgsonii* | | | | | | |
| Gene | Location | Exon I (bp) | Intron I (bp) | Exon II (bp) | Intron II (bp) | Exon III (bp) |
| *atpF* | LSC | 145 | 708 | 410 |  |  |
| *clpP* | LSC | 71 | 819 | 291 | 623 | 229 |
| *ndhA* | SSC | 553 | 1060 | 539 |  |  |
| *ndhB* | IR | 777 | 671 | 756 |  |  |
| *petB* | LSC | 6 | 762 | 642 |  |  |
| *petD* | LSC | 9 | 718 | 474 |  |  |
| *rpl16* | LSC | 9 | 1035 | 399 |  |  |
| *rpl2* | IR | 393 | 667 | 435 |  |  |
| *rpoC1* | LSC | 432 | 739 | 1641 |  |  |
| *rps12* | LSC | 114 | - | 232 | 530 | 26 |
| *rps16* | LSC | 41 | 840 | 214 |  |  |
| *trnA-UGC* | IR | 38 | 821 | 35 |  |  |
| *trnG-UCC* | LSC | 23 | 727 | 47 |  |  |
| *trnI-GAU* | IR | 42 | 772 | 35 |  |  |
| *trnK-UUU* | LSC | 37 | 2543 | 35 |  |  |
| *trnL-UAA* | LSC | 37 | 440 | 50 |  |  |
| *trnV-UAC* | LSC | 38 | 573 | 37 |  |  |
| *ycf3* | LSC | 125 | 697 | 229 | 740 | 153 |
| *L. jaluensis* | | | | | | |
| Gene | Location | Exon I (bp) | Intron I (bp) | Exon II (bp) | Intron II (bp) | Exon III (bp) |
| *atpF* | LSC | 145 | 708 | 410 |  |  |
| *clpP* | LSC | 71 | 818 | 291 | 620 | 229 |
| *ndhA* | SSC | 553 | 1063 | 539 |  |  |
| *ndhB* | IR | 777 | 671 | 756 |  |  |
| *petB* | LSC | 6 | 762 | 642 |  |  |
| *petD* | LSC | 9 | 718 | 474 |  |  |
| *rpl16* | LSC | 9 | 1028 | 399 |  |  |
| *rpl2* | IR | 393 | 667 | 435 |  |  |
| *rpoC1* | LSC | 432 | 732 | 1641 |  |  |
| *rps12* | LSC | 114 | - | 232 | 530 | 26 |
| *rps16* | LSC | 41 | 841 | 214 |  |  |
| *trnA-UGC* | IR | 38 | 821 | 35 |  |  |
| *trnG-UCC* | LSC | 23 | 727 | 47 |  |  |
| *trnI-GAU* | IR | 42 | 772 | 35 |  |  |
| *trnK-UUU* | LSC | 37 | 2556 | 35 |  |  |
| *trnL-UAA* | LSC | 37 | 440 | 50 |  |  |
| *trnV-UAC* | LSC | 38 | 573 | 37 |  |  |
| *ycf3* | LSC | 125 | 697 | 229 | 740 | 153 |
| *L. mongolica* | | | | | | |
| Gene | Location | Exon I (bp) | Intron I (bp) | Exon II (bp) | Intron II (bp) | Exon III (bp) |
| *atpF* | LSC | 145 | 708 | 410 |  |  |
| *clpP* | LSC | 71 | 818 | 291 | 620 | 229 |
| *ndhA* | SSC | 553 | 1062 | 539 |  |  |
| *ndhB* | IR | 777 | 671 | 756 |  |  |
| *petB* | LSC | 6 | 762 | 642 |  |  |
| *petD* | LSC | 9 | 718 | 474 |  |  |
| *rpl16* | LSC | 9 | 1035 | 399 |  |  |
| *rpl2* | IR | 393 | 667 | 435 |  |  |
| *rpoC1* | LSC | 432 | 731 | 1641 |  |  |
| *rps12* | LSC | 114 | - | 232 | 530 | 26 |
| *rps16* | LSC | 41 | 840 | 214 |  |  |
| *trnA-UGC* | IR | 38 | 821 | 35 |  |  |
| *trnG-UCC* | LSC | 23 | 727 | 47 |  |  |
| *trnI-GAU* | IR | 42 | 772 | 35 |  |  |
| *trnK-UUU* | LSC | 37 | 2556 | 35 |  |  |
| *trnL-UAA* | LSC | 37 | 440 | 50 |  |  |
| *trnV-UAC* | LSC | 38 | 573 | 37 |  |  |
| *ycf3* | LSC | 125 | 697 | 229 | 740 | 153 |
| *L. veitchiana* | | | | | | |
| Gene | Location | Exon I (bp) | Intron I (bp) | Exon II (bp) | Intron II (bp) | Exon III (bp) |
| *atpF* | LSC | 145 | 708 | 410 |  |  |
| *clpP* | LSC | 71 | 839 | 291 | 619 | 229 |
| *ndhA* | SSC | 553 | 1065 | 539 |  |  |
| *ndhB* | IR | 777 | 671 | 756 |  |  |
| *petB* | LSC | 6 | 772 | 642 |  |  |
| *petD* | LSC | 9 | 718 | 474 |  |  |
| *rpl16* | LSC | 9 | 1035 | 399 |  |  |
| *rpl2* | IR | 393 | 667 | 435 |  |  |
| *rpoC1* | LSC | 432 | 734 | 1641 |  |  |
| *rps12* | LSC | 114 | - | 232 | 530 | 26 |
| *rps16* | LSC | 41 | 840 | 214 |  |  |
| *trnA-UGC* | IR | 38 | 821 | 35 |  |  |
| *trnG-UCC* | LSC | 23 | 707 | 47 |  |  |
| *trnI-GAU* | IR | 42 | 772 | 35 |  |  |
| *trnK-UUU* | LSC | 37 | 2557 | 35 |  |  |
| *trnL-UAA* | LSC | 37 | 440 | 50 |  |  |
| *trnV-UAC* | LSC | 38 | 573 | 37 |  |  |
| *ycf3* | LSC | 125 | 696 | 229 | 740 | 153 |
| *L. fischeri* | | | | | | |
| Gene | Location | Exon I (bp) | Intron I (bp) | Exon II (bp) | Intron II (bp) | Exon III (bp) |
| *atpF* | LSC | 145 | 708 | 410 |  |  |
| *clpP* | LSC | 71 | 818 | 291 | 621 | 229 |
| *ndhA* | SSC | 553 | 1064 | 539 |  |  |
| *ndhB* | IR | 777 | 671 | 756 |  |  |
| *petB* | LSC | 6 | 762 | 642 |  |  |
| *petD* | LSC | 9 | 718 | 474 |  |  |
| *rpl16* | LSC | 9 | 1046 | 399 |  |  |
| *rpl2* | IR | 393 | 667 | 435 |  |  |
| *rpoC1* | LSC | 432 | 732 | 1641 |  |  |
| *rps12* | LSC | 114 | - | 232 | 530 | 26 |
| *rps16* | LSC | 41 | 841 | 214 |  |  |
| *trnA-UGC* | IR | 38 | 821 | 35 |  |  |
| *trnG-UCC* | LSC | 23 | 727 | 47 |  |  |
| *trnI-GAU* | IR | 42 | 772 | 35 |  |  |
| *trnK-UUU* | LSC | 37 | 2556 | 35 |  |  |
| *trnL-UAA* | LSC | 37 | 440 | 50 |  |  |
| *trnV-UAC* | LSC | 38 | 573 | 37 |  |  |
| *ycf3* | LSC | 125 | 697 | 229 | 740 | 153 |
