# Supplementary material for: Identification of Ligularia Herbs Using the Complete Chloroplast Genome as a Super-Barcode
Source: Front Pharmacol. 2018 Jul 3;9:695. doi: 10.3389/fphar.2018.00695 (PMC6043804; doi:10.3389/fphar.2018.00695)
Supplement: Supplementary file 6 [file Table_6.docx]

Supplementary Material

# TABLE S6 | Long repeat sequences in the CP genome of *L. hodgsonii*.

| ID | Repeat Start 1 | Type | Size (bp) | Repeat Start 2 | Mismatch (bp) | E-Value | Gene | Region |
| --- | --- | --- | --- | --- | --- | --- | --- | --- |
| 1 | 8009 | F | 30 | 8033 | -3 | 6.11E-04 | IGS | LSC |
| 2 | 46395 | F | 30 | 46416 | -2 | 2.18E-05 | IGS | LSC |
| 3 | 66504 | F | 30 | 98268 | -2 | 2.18E-05 | IGS | LSC; IRb |
| 4 | 90334 | F | 30 | 90352 | -3 | 6.11E-04 | *ycf2* (CDS) | IRb |
| 5 | 125528 | F | 30 | 125573 | -3 | 6.11E-04 | *ycf1* (CDS) | SSC |
| 6 | 125544 | F | 30 | 125589 | -3 | 6.11E-04 | *ycf1* (CDS) | SSC |
| 7 | 144007 | F | 30 | 144025 | -3 | 6.11E-04 | *ycf2* (CDS) | IRa |
| 8 | 90314 | F | 31 | 90341 | -3 | 1.69E-04 | *ycf2* (CDS) | IRb |
| 9 | 90314 | F | 31 | 90323 | -3 | 1.69E-04 | *ycf2* (CDS) | IRb |
| 10 | 144017 | F | 31 | 144044 | -3 | 1.69E-04 | *ycf2* (CDS) | IRa |
| 11 | 144035 | F | 31 | 144044 | -3 | 1.69E-04 | *ycf2* (CDS) | IRa |
| 12 | 8529 | F | 32 | 34906 | -3 | 4.66E-05 | IGS | LSC |
| 13 | 38088 | F | 32 | 40312 | -3 | 4.66E-05 | *psaB* (CDS); *psaA* (CDS) | LSC |
| 14 | 43114 | F | 35 | 94064 | -3 | 9.62E-07 | *ycf3* (CDS); *ndhB* (intron) | LSC; IRb |
| 15 | 94064 | F | 35 | 118455 | -3 | 9.62E-07 | *ndhB* (intron); *ndhA* (intron) | IRb; SSC |
| 16 | 43111 | F | 39 | 118452 | 0 | 2.13E-14 | *ycf3* (intron); *ndhA* (intron) | LSC; SSC |
| 17 | 97105 | F | 39 | 118452 | -1 | 2.49E-12 | IGS; *ndhA* (intron) | IRb; SSC |
| 18 | 43109 | F | 41 | 97103 | -1 | 1.63E-13 | *ycf3* (intron); IGS | LSC; IRb |
| 19 | 8531 | P | 30 | 44844 | -2 | 2.18E-05 | IGS; *trnS-GGA* (tRNA) | LSC |
| 20 | 34908 | P | 30 | 44844 | -1 | 5.02E-07 | IGS; *trnS-GGA* (tRNA) | LSC |
| 21 | 34966 | P | 30 | 44782 | -3 | 6.11E-04 | *trnS-UGA* (tRNA); IGS | LSC |
| 22 | 66504 | P | 30 | 136091 | -2 | 2.18E-05 | IGS | LSC; IRa |
| 23 | 90334 | P | 30 | 144007 | -3 | 6.11E-04 | *ycf2* (CDS) | IRb; IRa |
| 24 | 90352 | P | 30 | 144025 | -3 | 6.11E-04 | *ycf2* (CDS) | IRb; IRa |
| 25 | 30864 | P | 31 | 30864 | -3 | 1.69E-04 | IGS | LSC |
| 26 | 90314 | P | 31 | 144017 | -3 | 1.69E-04 | *ycf2* (CDS) | IRb; IRa |
| 27 | 90314 | P | 31 | 144035 | -3 | 1.69E-04 | *ycf2* (CDS) | IRb; IRa |
| 28 | 90323 | P | 31 | 144044 | -3 | 1.69E-04 | *ycf2* (CDS) | IRb; IRa |
| 29 | 90341 | P | 31 | 144044 | -3 | 1.69E-04 | *ycf2* (CDS) | IRb; IRa |
| 30 | 110565 | P | 31 | 110565 | -3 | 1.69E-04 | IGS | SSC |
| 31 | 118806 | P | 31 | 118815 | -3 | 1.69E-04 | *ndhA* (intron) | SSC |
| 32 | 117159 | P | 32 | 117159 | 0 | 3.48E-10 | IGS | SSC |
| 33 | 43114 | P | 35 | 140290 | -3 | 9.62E-07 | *ycf3* (intron); *ndhB* (CDS) | LSC; IRa |
| 34 | 118455 | P | 35 | 140290 | -3 | 9.62E-07 | *ndhA* (intron); *ndhB* (CDS) | SSC; IRa |
| 35 | 118452 | P | 39 | 137245 | -1 | 2.49E-12 | *ndhA* (intron); IGS | SSC; IRa |
| 36 | 43109 | P | 41 | 137245 | -1 | 1.63E-13 | *ycf3* (intron); IGS | LSC; IRa |
| 37 | 10645 | P | 46 | 10645 | -2 | 1.21E-14 | IGS | LSC |
| 38 | 73325 | P | 48 | 73325 | 0 | 8.11E-20 | IGS | LSC |
| 39 | 66862 | R | 31 | 66868 | -3 | 1.69E-04 | IGS | LSC |

F: Forward; P: palindromic; R: reverse; IGS: intergenic space; CDS: protein-coding regions.
